# Ank-mediated pyrophosphate regulates shear stress-induced small extracellular vesicle production in 3D-cultured osteocytes

**DOI:** 10.1080/19768354.2024.2409460

**Published:** 2024-10-22

**Authors:** Su Jeong Lee, Deuk Kju Jung, Soomin Im, Changkook You, Jung-Eun Kim, Jong-Sup Bae, Mee-seon Kim, Kyungmoo Yea, Eui Kyun Park

**Affiliations:** a Department of Oral Pathology and Regenerative Medicine, School of Dentistry, Institute for Hard Tissue and Bio-tooth Regeneration (IHBR), Kyungpook National University, Daegu, Republic of Korea; b Department of Molecular Medicine, Cell and Matrix Research Institute (CMRI), School of Medicine, Kyungpook National University, Daegu, Republic of Korea; c College of Pharmacy, CMRI, Research Institute of Pharmaceutical Sciences, Kyungpook National University, Daegu, Republic of Korea; d Department of New Biology, Daegu Gyeongbuk Institute of Science and Technology, Daegu, Republic of Korea

**Keywords:** Osteocyte, small extracellular vesicles, shear stress, Ank, BCP scaffold

## Abstract

Osteocytes are located in the lacunae of fluid-filled bone and communicate with neighboring or distant cells by secreting small extracellular vesicles (sEVs) and growth factors as well as via dendrite–dendrite direct connections. However, the mechanism regulating sEV production in osteocytes is yet to be elucidated. In this study, we investigated sEV production and its underlying mechanism in osteocytes cultured on a three dimensional (3D) scaffold. We employed a perfusion system to apply shear stress stimulation to MLO-Y4 cells cultured on a 3D biphasic calcium phosphate (BCP) scaffold and analyzed sEV production and gene expression using RNA sequencing. We found that the expression of genes associated with sEV biogenesis and the secretory pathway were enhanced by fluid shear stress in MLO-Y4 cells cultured on a 3D BCP scaffold. In particular, fluid shear stress induced the expression of Ank, a pyrophosphate transporter, in 3D-cultured MLO-Y4 cells. The role of Ank in sEV production was further examined. Probenecid, an Ank inhibitor, significantly suppressed shear stress-induced sEV production, whereas Ank cDNA overexpression stimulated it. The inhibition of shear stress-induced sEV production by probenecid was recovered by the exogenous addition of pyrophosphate to MLO-Y4 cells. These findings suggest that shear stress-mediated sEV production in 3D-cultured osteocytes is regulated by extracellular pyrophosphate transported by Ank.

## Introduction

Osteocytes, one of the major bone cell types, are deeply embedded in the mineralized bone matrix spaces as known as lacunae (Dallas et al. 2013). Despite their enclosed environment, osteocytes reportedly establish an extensive and highly connected syncytial network characterized by small dendritic processes extending through tiny channels called canaliculi (Matsuo 2009). These processes connect with other osteocytes, osteoblasts, and cells on the bone surface, creating a vast communication network. This cell-to-cell communication is facilitated by the flow of interstitial fluid through the bone pores and canaliculi, transmitting signals between osteocytes and other bone cells. Osteocytes act as sensors sensitive to fluid shear stress and utilize these biophysical signals to release molecules that modulate bone metabolism, including bone remodeling (Cheng et al. 2001; Cherian et al. 2005; Kulkarni et al. 2010; Li et al. 2012; Spatz et al. 2015). The intercellular communication within the bone microenvironment is extended beyond interactions solely between bone cells. Notably, osteocytes have recently been recognized to possess the ability to regulate the function of distant organs by secreting endocrine factors and signaling transmitters (Zaidi et al. 2023). In other words, osteocyte-derived biologically active molecules, including cytokines, growth factors, and small extracellular vesicles (sEVs), can serve as effective mediators in facilitating communication between osteocytes and other tissues (Shimada et al. 2004; Gutiérrez et al. 2011; Sato et al. 2017; Sano et al. 2021; Dong et al. 2022; Cheng et al. 2023).

sEVs constitute a heterogeneous group of membrane-structured vesicles that are released into the extracellular environment by almost all living cell types (Raposo and Stoorvogel 2013; van Niel et al. 2018). According to the minimal information for studies of extracellular vesicles (MISEV2023) guidelines, sEVs are usually identified as having a diameter < 200 nm, containing diverse cellular components, including nucleic acids, proteins, lipids, and metabolites (Gomzikova et al. 2021; Yokoi and Ochiya 2021). They play important roles as intercellular communication mediators to transfer bioactive components from donor to recipient cells, resulting in the alteration of their physiological and pathological functions (Valadi et al. 2007; Milane et al. 2015; Pegtel and Gould 2019). Various types of mechanical stimulation can regulate the crosstalk between cells and their microenvironment, potentially leading to sEV secretion for communication (Turturici et al. 2014). Based on this rationale, several studies have reported that mechanical stimulation enhances sEV secretion. In human dental pulp stem cells and mesenchymal stem cells, direct flow stimulation at rates of 0.5 and 1.0 mL/min increased sEV production, respectively (Guo et al. 2021). Additionally, sEV production in human dermal microvascular endothelial cells increased at a flow rate at 1.5 × 10^−2^ dyn/cm^2^ (Patel et al. 2019). In particular, leveraging the responsiveness of osteocytes to fluid shear stimulation reportedly promotes sEV secretion (Morrell et al. 2018). However, the mechanism by which flow stimulation to cells induces sEV secretion remains unclear.

Therefore, we focused on the mechanism behind sEV production under biocompatible conditions by employing a perfusion culture system that applies shear stress stimulation to osteocytes cultured on a 3D biphasic calcium phosphate (BCP) scaffold. In the present study, we newly identified gene expression alterations triggered by the shear stress response in BCP scaffold-based osteocytes by comprehensively analyzing the osteocyte transcriptome, thereby advancing the molecular mechanism governing sEV secretion in 3D osteocytes.

## Materials and methods

### Manufacture of 3D BCP scaffolds

Interconnected macro- and microporous scaffolds can be achieved using the sponge replication method, where calcium phosphate paste is coated on the strut of a polyurethane sponge and sintered at an elevated temperature, followed by additional slurry coating on the sintered body and further sintering. BCP powder, comprising 60% hydroxyapatite and 40% β-tricalcium phosphate, was ultrasonically dispersed in 1% polyvinyl alcohol aqueous binder solution with 0.2% of Darvan®-CN as a dispersant, and the powder/binder solution weight ratio was set to 1.4/1. The prepared BCP paste was coated on 60-ppi (pores per inch) polyurethane sponge disc blocks of 30-mm diameter and 3-mm thickness via repetitive manual tapping in consideration of shrinkage after sintering. As-coated scaffolds were dried at 40℃ for 1 h and 100℃ for 30 min, respectively, and sintered at 1,200℃ for 3 h at a heating rate of 2℃/min (from 150 and 400℃ to 600℃ at 3℃/min and 1,200℃ at 5℃/min, respectively). For the second coating, BCP slurry with 10% powder was prepared via ultrasonic dispersion in 0.6% polyvinyl alcohol aqueous binder. Sintered BCP blocks were immersed in slurry, and excess slurry within a scaffold was removed by gently blowing air and subsequently absorbing it on a paper tissue. The slurry-coated scaffolds were dried at 60℃ for 1 h and sintered at 1,150℃ for 2 h at a heating rate of 5℃/min for microporosity on the surface of the struts. The sintered scaffolds were machined into a 20-mm diameter and 2-mm thickness on the 15-μm diamond plate of a grinding polisher. Finally, the BCP scaffolds were ultrasonically washed twice in distilled water and subsequently in ethanol; thereafter, they were dried at 80℃ for 1 h.

### Cell culture and shear stress applications

MLO-Y4 osteocyte-like cells were cultured on a collagen-coated plate (rat-tail collagen type I, 0.15 mg/mL in 0.02 N acetic acid solution) in alpha minimum essential medium (Gibco, Grand Island, NY, USA) containing 5% fetal bovine serum (HyClone™, Logan, UT, USA) and 5% calf serum (Gibco) and grown at 37℃ in a 5% CO_2_ humidified air incubator.

**Figure 1.**
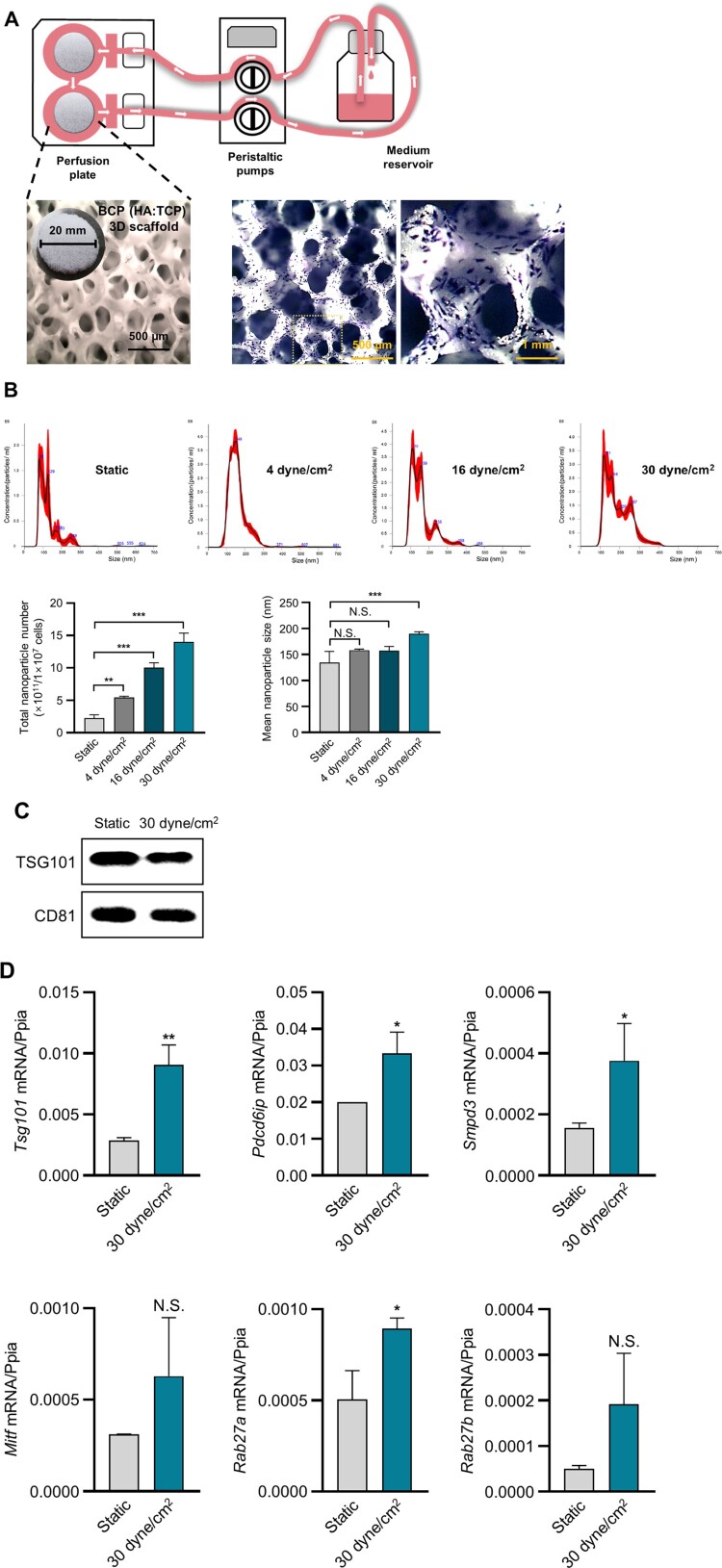
Characteristics of sEVs derived from 3D BCP scaffold-cultured MLO-Y4 cells. (A) Schematic illustration of perfusion system (top), and images showing an BCP scaffold (left) and crystal violet staining of MLO-Y4 cells growing on BCP scaffold (right). (B) Particle size, concentration, and total nanoparticle number of sEVs from scaffold-cultured osteocytes under different stress conditions according to NTA. One-way ANOVA multiple comparison test. *p < 0.05, **p < 0.01, ***p < 0.001. (C) Western blotting of TSG101 and CD81 in the isolated sEVs. (D) mRNA expression levels of genes in the sEV production-related pathway according to real-time PCR. * *p *< 0.05, *t* test

To prepare the 3D MLO-Y4 culture subjected to fluid flow shear stress, MLO-Y4 cells were treated with trypsin and seeded on BCP scaffold, and cultured in an Alvetex® perfusion plate (AVP011; Reprocell, Yokohama, Japan). Cell suspension (400 µL containing (2.0 × 10^6^–2.5 × 10^6^ cells/BCP scaffold)) was applied to prevent it from seeping underneath the scaffold, and adequate time (3 h) was provided for the cell to infiltrate the scaffold internal structure. Once cell adherence was complete, the fluid perfusion dispenser (BT101F; Lead Fluid Technology Co., Ltd., Baoding, China) was adjusted to a shear stress range of 4–30 dyne/cm^2^, allowing a continuous flow of cell culture media through the perfusion culture for 24 h (Figure 1(a)).

BCP scaffold-bound MLO-Y4 cells were stained using crystal violet solution (0.1% w/v in 20% methanol). The cell morphology was then visualized using an Olympus SZX9 microscope.

MLO-Y4 cells were pretreated with 50 μM Probenecid (P8761; Sigma-Aldrich, Saint Louis, MO, USA) in exosome-depleted medium while under fluid flow shear stress. Thereafter, the MLO-Y4 cells were treated with 10 nM sodium pyrophosphate tetrabasic (P8010, Sigma-Aldrich) 1 h after initiating the perfusion culture.

### sEV isolation and characterization

sEVs were isolated from conditioned media using sEV-depleted serum in each group. The media were filtered using a 0.2-μm membrane filter to remove cells and debris. For the ultrafiltration-based purification of sEVs, the filtered media were concentrated at 3,000 g and 4℃ for 30 min using a 100-kDa molecular weight cutoff filter (Pall Corporation, Port Washington, NY, USA) and subsequently incubated with a 1/5 volume of ExoQuick-TC™ reagent (ExoTC50A-1, SBI, Palo Alto, CA, USA) at 4℃ overnight. The reaction mixture was centrifuged at 1,500 g for 30 min to remove any trace of supernatant. The purified sEVs were stored at −80℃ in a freezer until use. sEV size and number were measured using a nanoparticle tracking analysis (NTA) system (NanoSight NS 300, Malvern Panalytical Ltd., Worcestershire, UK).

### Cytotoxicity assay

Drug concentration was optimized using Cell Counting Kit-8 (CCK-8, CK04; Dojindo Laboratories, Kumamoto, Japan), as described previously (Chen et al. 2022). Briefly, MLO-Y4 cells were seeded at a density of 6 × 10^3^ cell/well in 96-well plates and treated with graded concentrations of probenecid for 24 h. CCK-8 solution (10 µL) was added to each well of the plate after the experimental period, followed by additional incubation for 2 h at 37℃. Absorbance was measured at 450 nm using a microplate reader (Bio-Rad, Hercules, CA, USA).

### RNA isolation and quantitative real-time polymerase chain reaction (qRT-PCR)

Total RNA was extracted using TRIzol™ reagent (Bio Science Technology, Daegu, Korea) according to the manufacturer’s protocol. cDNA was synthesized with 1 μg of total RNA using SuperScript™ II reverse transcriptase (Invitrogen, Carlsbad, CA, USA). qRT-PCR was performed using SYBR® Master Mix (Takara Bio, Inc., Shiga, Japan) in a LightCycler® 1.5 (Roche Diagnostics, Basel, Switzerland) as described previously (Lim et al. 2023). Peptidylprolyl isomerase A (*Ppia*) was used as a reference gene. The primer sequences of the genes are shown in Table 1.

**Table 1.** Primer sequences.

Gene	Sequence (5′ – 3′)
Tsg101_F	GGTTATCCTGGCTGTCCTTACC
Tsg101_R	GCACGGATAGTGTCCTCACTGA
Pdcd6ip_F	GGACTCCATCCAATGACCTGTAC
Pdcd6ip_R	CGGCTTACACAGAAGTGCGATG
Smpd3_F	TCAACAGCGGTCTCTTCTTCGC
Smpd3_R	CTTTGGTCCTGAGGTGTGCTTC
Mitf_F	GATCGACCTCTACAGCAACCAG
Mitf_R	GCTCTTGCTTCAGACTCTGTGG
Rab27a_F	GAGCAAAGTTTCCTCAATGTCCG
Rab27a_R	CTTTCACTGCCCTCTGGTCTTC
Rab27b_F	CCTCACCAGTCAACAGAGCTTC
Rab27b_R	GCCGTTCATTGACTTCCCTTTGG
Nqo1_F	GCCGAACACAAGAAGCTGGAAG
Nqo1_R	GGCAAATCCTGCTACGAGCACT
Msmo1_F	CTGTGCAGTCATTGAGGACACC
Msmo1_R	GGGTTTCCAAGGGATGTGCGTA
Ank_F	CGTGGACTCATGCTGGCATTCT
Ank_R	GTTCTCGGCATTCCAGGTGACT
Nsdhl_F	TTAACCGCAGCCATTCGTCCTC
Nsdhl_R	GGTGAAGTCCACCAGGTTTTCC
Cyp51_F	ATCCAGAAGCGCAGGCTGTCAA
Cyp51_R	CAGTCCGATGAGCATCCCTGAT
Dhcr24_F	CTGGAGAACCACTTCGTGGAAG
Dhcr24_R	CTCCACATGCTTGAAGAACCAGG
Mvd_F	CAGCTAGTCCACCGCTTCAACA
Mvd_R	CAAACTCAGCCACAGTGTCCTC
Ppia_F	CATACAGGTCCTGGCATCTTGTC
Ppia_R	AGACCACATGCTTGCCATCCAG

### RNA-sequencing analysis

To identify differentially expressed genes between static and shear stress (30 dyne/cm^2^) samples, total RNA was isolated using TRIzol reagent (Invitrogen). Gene expression profiles were obtained via total RNA sequencing services provided by a specialized service provider (Ebiogen, Inc., Seoul, Korea). Gene classification was based on searches performed on the DAVID (http://david.abcc.ncifcrf.gov/) and Medline (http://www.ncbi.nlm.nih.gov/) databases. Data mining was performed using Excel-Based Differentially Expressed Gene Analysis software (ExDEGA, version 4.0.3; Ebiogen, Inc.). The genetic network of the differentially expressed genes was mapped using Cytoscape (version 3.10.1; https://cytoscape.org/index.html), an open-source software platform. Experimental data were integrated and analyzed using ClueGO (http://apps.cytoscape.org/apps/cluego), a plug-in app of Cytoscape, and functional analysis was performed using gene ontology (GO).

### Statistical analysis

Data were analyzed using GraphPad Prism (GraphPad Software, Inc.) and are expressed as the mean ± standard deviation. Statistical significance was determined using the two-tailed Student’s *t* test and one-way analysis of variance. Statistical significance was set at *p* < 0.05, <0.01, or <0.001.

## Results

### Fluid shear stress-stimulated sEV production in MLO-Y4 cells cultured on a 3D BCP scaffold

To examine sEV production in fluid shear stress-stimulated MLO-Y4 cells cultured on a 3D BCP scaffold, an *in vitro* model was established (Figure 1(a)). After confirming that the cells were evenly and stably seeded on the scaffold, as shown in Figure 1(a), we assessed the effect of increasing flow rates on MLO-Y4-derived sEV production. We collected the media from MLO-Y4 cells stimulated with or without fluid shear stress. The medium for MLO-Y4 cells without fluid shear stress was referred to as static, while that for MLO-Y4 cells with fluid shear stress was referred to as shear stress (4–30 dyne/cm^2^). The examination of isolated sEVs using an NTA system indicated that sEV production in MLO-Y4 cells cultured on a 3D BCP scaffold under fluid shear stress (4, 16 and 30 dyne/cm^2^) was 2.4-, 4.4-, and 6.2-fold higher than that in the 3D static group (Figure 1(b)). The mean diameter of the sEVs was approximately <200 nm (Figure 1(b)). Hence, we selected the 30 dyne/cm^2^ group, which exhibited the highest sEV production, as the representative sample, and confirmed the presence of sEV-surface-marker proteins, TSG101 and CD81 using Western blotting (Figure 1(c)). The sequential process from sEV production to secretion can be divided into three steps: sEV biogenesis, multivesicular body (MVB) transportation to the plasma membrane, and fusion with the plasma membrane. sEVs are generated from the invagination of the endosomal membrane, leading to the formation of intraluminal vesicles (ILVs) within large MVBs. Thereafter, the MVBs migrate toward and fuse with the plasma membrane before releasing sEVs into the extracellular environment. ILV generation in MVBs involves multiple molecular mechanisms, including the endosomal sorting complex required for transport (ESCRT)-dependent (*Tsg101* and *Alix*) and ESCRT-independent (*Smpd3*) pathways. The Rab family of small GTPases modulates vesicle trafficking, fusion with the plasma membrane, and sEV secretion (Hessvik and Llorente 2018; van Niel et al. 2018; Kalluri and LeBleu 2020; Kita and Shimomura 2022). To investigate the effect of fluid shear stress on the expression of genes involved in sEV biogenesis and secretion in MLO-Y4 cells cultured on a 3D BCP scaffold, we examined the expression of those genes, *Tsg101, Alix, Smpd3*, and *Rab27a*, markedly increased in MLO-Y4 cells under 30 dyne/cm^2^ fluid shear stress. In contrast, no significant differences in the expression of *Mitf* or *Rab27b* were observed in MLO-Y4 cells under 30 dyne/cm^2^ fluid shear stress compared with those in MLO-Y4 cells under static conditions (Figure 1(d)). These results suggest that sEV secretion from MLO-Y4 cells cultured on a 3D BCP scaffold was stimulated by shear stress, and the mRNA expression of genes involved in sEV biogenesis and its secretory pathway was significantly upregulated.

### Transcriptomic analysis in MLO-Y4 cells cultured on 3D BCP scaffold in response to fluid shear stress

**Figure 2.**
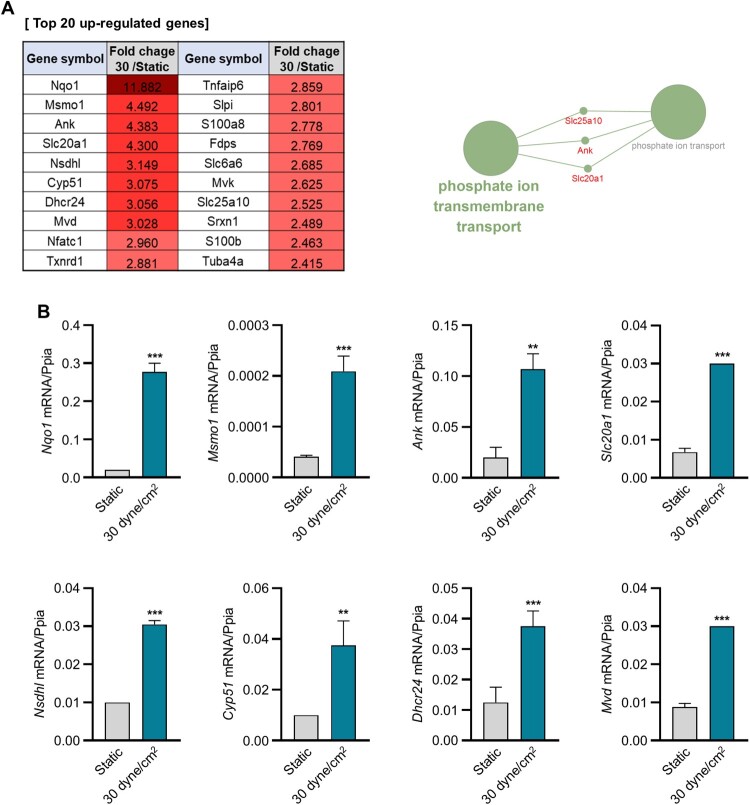
Gene expression analysis via transcriptomic and real-time PCR analyses of 3D-cultured MLO-Y4 cells in response to fluid shear stress. (A) Top 20 upregulated genes according to transcriptomic analysis of 3D-cultured MLO-Y4 cells in response to fluid shear stress (left). Cytoscape 3.10.1 visualization of the GO pathways of differentially expressed genes using ClueGo v.2.5.10 (right). (B) mRNA expression levels of differentially expressed genes according to real-time PCR. **p < 0.01, ***p < 0.001 versus the expression level of *Ppia*, *t* test

To elucidate the gene expression profile induced by fluid shear stress, we performed RNA sequencing of MLO-Y4 cells cultured on a 3D BCP scaffold. The cells were subjected to 30 dyne/cm^2^ fluid shear stress, and their gene expression was compared to that of cells cultured under static conditions within the same 3D environment. We analyzed genes exhibiting increased expression in MLO-Y4 cells stimulated with 30 dyne/cm^2^ fluid shear stress in comparison to those cultured under static conditions within the same 3D environment. We subsequently extracted 45 genes whose normalized expression values exceeded 10 under 30 dyne/cm^2^ fluid shear stress, and these values were 2.0-fold greater than those generated under static conditions. The top 20 differentially upregulated genes in cells subjected to 30 dyne/cm^2^ fluid shear stress compared to those under static conditions are shown in Figure 2(a). The results revealed that the upregulated genes were those involved in cholesterol biosynthesis, antioxidant activity, and phosphate ion transmembrane transport. The top 20 upregulated genes were validated using qRT-PCR (Figure 2(b)). *Nqo1*, *Msmo1*, *Ank*, *Slc20a1*, *Nsdhl*, *Cyp51*, *Dhcr24*, and *Mvd* were significantly upregulated in cells stimulated with 30 dyne/cm^2^ fluid shear stress compared with those in cells under static conditions. Based on recent studies indicating the involvement of cholesterol in sEV biogenesis and release (Boura et al. 2012; Abdullah et al. 2021), we subsequently focused on phosphate ion transmembrane transport, specifically emphasizing Ank, which exhibited the highest expression levels.

### ANK altered sEV production in 3D BCP scaffold-cultured MLO-Y4 cells in response to fluid shear stress

**Figure 3.**
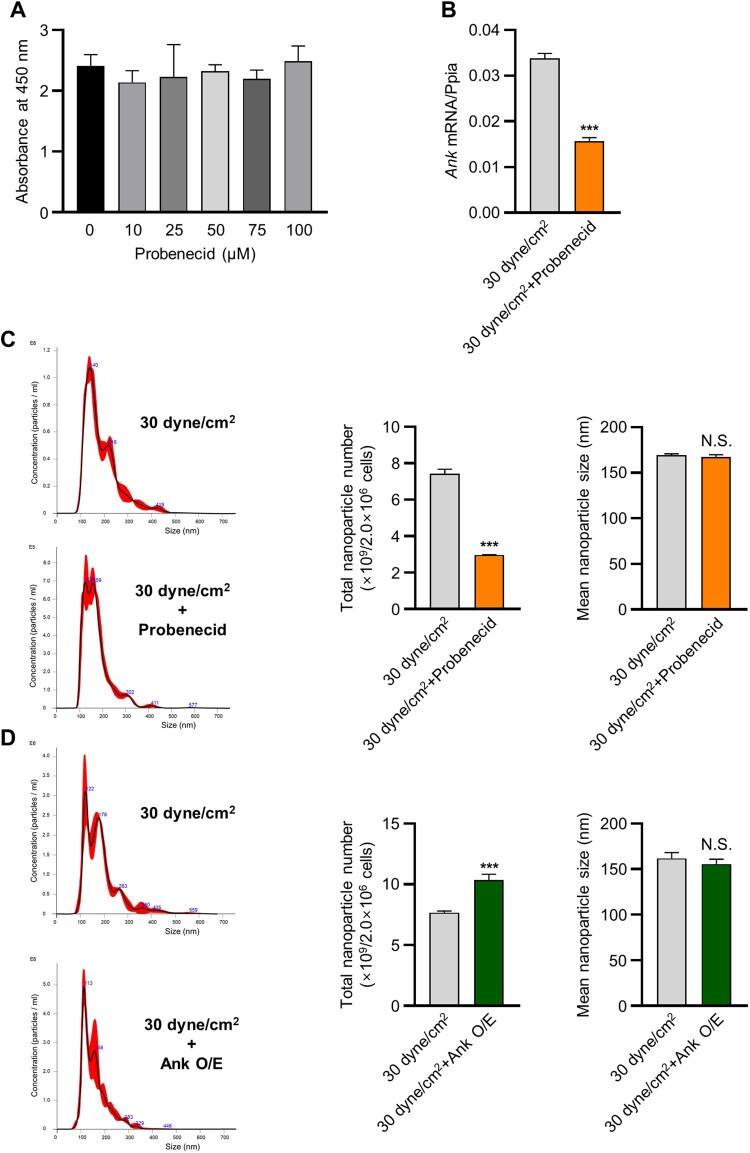
ANK-altered sEV production in 3D BCP scaffold-cultured MLO-Y4 cells subjected to fluid shear stress. (A) Cell viability was assessed using the CCK-8 assay at various doses of probenecid (0–100 μM). (B) mRNA expression level of *ANK* in 3D-cultured MLO-Y4 cells treated with DMSO or probenecid (Ank inhibitor) under 30 dyne/cm^2^ fluid shear stress according to real-time PCR. Total nanoparticle number and size from 3D-cultured MLO-Y4 cells (C) treated with DMSO or probenecid and (D) transfected with Ank plasmid DNA under 30 dyne/cm^2^ fluid shear stress according to NTA analysis. ***p < 0.001, *t* test.

Based on the results indicating ANK upregulation under 30 dyne/cm^2^ fluid shear stress, we confirmed whether ANK expression is sufficient to alter sEV secretion. MLO-Y4 cells were treated with the ANK inhibitor, probenecid, at concentrations between 10 and 50 µM (Figure 3(a)), and the CCK-8 assay was performed to examine cytotoxicity. The results revealed that probenecid exhibited no cytotoxicity. *Ank* expression was significantly inhibited under 30 dyne/cm^2^ fluid shear stress with probenecid compared with that in the control (Figure 3(b)). The NTA-based examination of isolated sEVs indicated that sEV production in probenecid-treated MLO-Y4 cells cultured on a 3D BCP scaffold under 30 dyne/cm^2^ fluid shear stress was lower than that in those without probenecid (Figure 3(c)). The mean sEV diameter displayed no significant difference between the two groups (<200 nm) (Figure 3(c)). We also observed *Ank* overexpression to markedly increase sEV production in MLO-Y4 cells cultured on a 3D BCP scaffold under 30 dyne/cm^2^ fluid shear stress, and the sEVs derived from each of the two groups exhibited no significant difference (< 200 nm) (Figure 3(d)).

### *In vitro* pyrophosphate influenced sEV production from 3D BCP scaffold-cultured MLO-Y4 cells under fluid shear stress conditions

**Figure 4.**
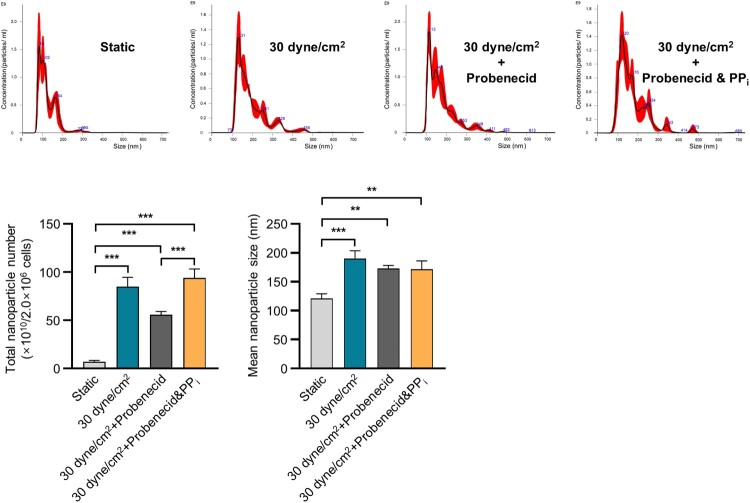
*In vitro* pyrophosphate (PP_i_) affected sEV production in 3D-cultured MLO-Y4 cells under fluid shear stress. The total nanoparticle number and size of 3D-cultured MLO-Y4 cells were evaluated under four conditions: static, 30 dyne/cm^2^ fluid shear stress, 30 dyne/cm^2^ fluid shear stress with probenecid, and 30 dyne/cm^2^ fluid shear stress with probenecid + 10 nM PP_i_ according to NTA analysis. **p < 0.01, ***p < 0.001, one-way ANOVA multiple comparison test.

ANK-induced sEV release suggests a potential link to changes in pyrophosphate concentration between intra- and extracellular environments. ANK serves as a transport channel, facilitating the movement of intracellular inorganic pyrophosphate (PP_i_) to extracellular compartments (Ho et al. 2000). Therefore, we focused on ANK’s cellular functions and investigated whether altering the concentration of extracellular pyrophosphate affects sEV secretion under 30 dyne/cm^2^ fluid shear stress. We used NTA to analyze sEV production under four conditions: static, 30 dyne/cm^2^ fluid shear stress, 30 dyne/cm^2^ fluid shear stress with probenecid, and 30 dyne/cm^2^ fluid shear stress with probenecid + 10 nM PP_i_ in cells cultured on a BCP scaffold. The mean sEV diameter was approximately < 200 nm (Figure 4). Interestingly, under conditions where probenecid inhibited Ank expression, the addition of 10 nM PP_i_ under 30 dyne/cm^2^ fluid shear stress led to a notable recovery in sEV production (Figure 4). Our findings suggest that fluid shear stress upregulates Ank expression, resulting in elevated extracellular PP_i_ levels and subsequently stimulating sEV production in MLO-Y4 cells cultured on a 3D BCP scaffold under fluid shear stress conditions.

## Discussion

Osteocytes, trapped in lacunae within the mineralized bone matrix, act as pivotal regulators in not only maintaining bone homeostasis but also orchestrating communication with neighboring cells and distant organs, thus contributing to systemic physiological coordination. sEVs have emerged as key mediators in this intercellular dialogue, facilitating the transport of bioactive molecules across cellular boundaries. Despite considerable attention to the composition and functions of osteocyte-derived sEVs in recent studies, investigations into the mechanism underlying sEV production in osteocytes remain relatively limited. Therefore, this study aimed to investigate sEV production and its underlying mechanisms in osteocytes cultured on a 3D scaffold.

To apply shear stress to the 3D culture system, we modified the Alvetex® perfusion system using a 3D BCP scaffold and analyzed sEV production. Scaffolds, typically characterized by their porous network structure, demonstrate functional utility exclusively within a bioreactor perfusion culture system, enabling the free permeation of biological fluids, including cell culture media, thereby creating advantageous conditions for promoting biological activity (Zhang et al. 2006; Yan et al. 2018). Specifically, BCP scaffolds, comprising HAp and TCP, represent an optimal bone substitute, fostering a microenvironment closely mirroring the *in vivo* settings for the MLO-Y4 cells employed in this investigation. Furthermore, these 3D cell culture systems can facilitate efficient sEV production compared with traditional 2D cell culture systems (Rocha et al. 2019; Thippabhotla et al. 2019). We compared sEV production from osteocyte cultured on 2D plate surface with those cultured on a 3D BCP scaffold. Our results showed that sEV production on the 3D BCP scaffold was 26% higher than on the 2D plate (data not shown). Owing to their embedded position within the mineralized matrix, osteocytes pose significant challenges to fully elucidating their responses to mechanical loading *in vivo*, rendering the precise physiological flow rate difficult to determine. However, theoretical models estimate that osteocytes experience fluid shear stress within the 8–30 dyne/cm^2^ range (Weinbaum et al. 1994). Based on this estimation, we analyzed the level of sEV production in cells subjected to fluid shear stress of up to 30 dyne/cm^2^. Our findings indicate that sEV production in MLO-Y4 cells cultured on a 3D BCP scaffold under fluid shear stress increased in a flow rate-dependent manner (4–30 dyne/cm^2^) compared with that in the static group (without fluid shear stress). Additionally, we observed an enhanced expression of genes associated with sEV biogenesis and the related secretory pathway in the 30 dyne/cm^2^ group compared with that in the static group. These results suggest that the 3D culture system supports the stable growth of numerous cells capable of producing sEVs, while fluid shear stress application significantly enhances sEV production.

We also examined RNA sequencing to analyze transcriptomic changes in MLO-Y4 cells cultured on a 3D BCP scaffold in response to 30 dyne/cm^2^ fluid shear stress, where the highest level of sEV production was observed. The transcriptome results revealed the highly upregulated expression of genes involved in cholesterol biosynthesis, as previously reported (Boura et al. 2012; Kulshreshtha et al. 2019; Abdullah et al. 2021), indicating our data’s high fidelity. In addition, we focused on the *Ank* gene, which is involved in phosphate ion transmembrane transport. We inquired whether *Ank* expression affects sEV production. We observed a significant decrease in sEV production when Ank expression was inhibited by probenecid, an Ank inhibitor. Conversely, the overexpression of Ank resulted in a notable increase in sEV production. Previous studies have also demonstrated that, in addition to genes directly involved in sEV biogenesis or the secretory pathway, several factors, such as mTORC1, which regulates autophagy, and cortactin, a cytoskeletal protein, potentially play a role in sEV secretion (Sinha et al. 2016; Zou et al. 2019). Therefore, these findings suggest that the expression level of Ank influences sEV production, simultaneously raising the question of whether this effect is mediated by Ank-induced cellular PP_i_ concentration differences.

Ank is primarily located in the plasma membrane and acts as a regulator, facilitating PP_i_ transport from the cytoplasm to the extracellular milieu. Previous studies have demonstrated that in Ank-mutant mice, intracellular PP_i_ levels increase, whereas extracellular PP_i_ levels decrease (Ho et al. 2000). Additionally, Ank expression is reportedly upregulated in the cartilage of degenerative osteoarthritis, where mineralization occurs (Johnson and Terkeltaub 2004). Therefore, we investigated whether PP_i_ translocation by ANK directly affects sEV production. ANK inhibition by probenecid followed by PP_i_ addition led to the recovery of sEV production. These results indicate that an increase in extracellular PP_i_ concentration in response to ANK promotes sEV production. However, concluding that a higher extracellular PP_i_ concentration than the intracellular level increases sEV production is challenging. Within our fluid shear stress system, shear stress-exposed MLO-Y4 cells exhibited upregulated ANK expression, resulting in the accumulation of extracellular PP_i_. This accumulation likely serves as a signaling mechanism in response to environmental changes, thereby promoting sEV production. Therefore, while the accumulation of PPi may partially influence sEV production, additional experiments are required to fully elucidate its role in this process. It is also noteworthy that, although our current findings do not address all aspects, sEVs from mechanically stimulated osteocytes have been reported to have supportive effects on various tissues (Shen et al. 2023; Chen et al. 2024; Zhu et al. 2024). Additionally, there is evidence suggesting that osteocyte-derived sEVs may offer protective effects against Alzheimer’s disease (Jiang et al. 2022). These observations imply that sEVs generated under shear stress could potentially circulate throughout the body via the bloodstream, contributing to tissues homeostasis. Therefore, further research is necessary to investigate how sEVs from osteocytes might influence bone remodeling and function, as well as their broader impact on overall tissue health. In summary, our study provides evidence that the pyrophosphate transporter ANK plays a critical role in sEV production under fluid shear stress conditions in MLO-Y4 cells cultured on a 3D BCP scaffold.

## Declaration of interest statement

The authors report no declarations of interest.
